# Dominance of attentional focus: a comparative study on its impact on standing postural control in healthy younger and older adults

**DOI:** 10.3389/fnhum.2024.1384305

**Published:** 2024-06-26

**Authors:** Shun Sawai, Shin Murata, Yuya Sakano, Shoya Fujikawa, Ryosuke Yamamoto, Yusuke Shizuka, Hideki Nakano

**Affiliations:** ^1^Graduate School of Health Sciences, Kyoto Tachibana University, Kyoto, Japan; ^2^Department of Rehabilitation, Kyoto Kuno Hospital, Kyoto, Japan; ^3^Department of Physical Therapy, Faculty of Health Sciences, Kyoto Tachibana University, Kyoto, Japan; ^4^Kissho-Home of Social Welfare Corporation Seiwaen, Kyoto, Japan; ^5^Department of Rehabilitation, Tesseikai Neurosurgical Hospital, Shijonawate, Japan

**Keywords:** attentional focus, dominance, older adults, standing postural control, attentional function

## Abstract

**Introduction:**

Attentional focus is a phenomenon in which shifting the focus of attention alters performance of standing postural control. It can be categorized as internal focus (IF), which directs attention to the body parts, or external focus (EF), which directs attention to the external environment. Although attentional focus that improves standing postural control in younger people exhibits individual dominance, the dominance of attentional focus in standing postural control in older adults remains ambiguous. Therefore, this study aimed to compare the dominance of attentional focus in standing postural control between healthy younger and older adults, a crucial step for understanding the aging process.

**Methods:**

The participants performed a standing postural control task under the IF and EF conditions. Based on the condition during which they exhibited superior performance, the participants were divided into two groups: IF-dominant and EF-dominant. The standing postural control performance in each group under the IF and EF conditions was subsequently compared.

**Results:**

The results showed that the participants, encompassing both younger and older adults, were divided into the IF-dominant and EF-dominant groups, confirming the dominance of attentional focus. The performance under the EF condition in older adults was also influenced by the dominance of attentional focus.

**Conclusion:**

These results highlight the potential importance of intervention methods based on the dominance of attentional focus, providing valuable insights into future research and clinical practice.

## 1 Introduction

Falls among older adults, hindering their health and increasing the economic burden on society, pose a significant public health problem (World Health Organization, 2021). In recent years, particularly with the global aging of the population, the number of older adults who experience falls has been increasing, necessitating proactive preventive interventions (Pinheiro et al., 2022; Salari et al., 2022). Impaired standing balance is a major risk factor for falls. Therefore, the effectiveness of exercise interventions in improving balance disorders has been extensively investigated (Granacher et al., 2011; Lesinski et al., 2015; Loureiro et al., 2021). Perturbation-based balance training that induces reactive balance control has recently been reported to improve standing postural control ability (Gerards et al., 2017). Furthermore, advancements in science and technology have led to the development of new intervention methods, such as virtual reality (Chen et al., 2021) and vision-related trainings (Mak et al., 2021), aimed at improving the effectiveness of exercise interventions. Consequently, there is ongoing development and refinement of new fall prevention strategies for older adults. Continuing to explore additional methods to mitigate the risk of falls in this demographic is imperative.

A decline in attentional function has been observed to have a negative influence on standing balance (Woollacott and Shumway-Cook, 2002). Compared with younger adults, older adults' standing postural control performance is reduced by attentional cost demands, such as dual tasks (Maylor and Wing, 1996; Boisgontier et al., 2013). This suggests that standing postural control in older adults requires more attentional resources and reduced attentional function increases postural sway.

Performance varies depending on the focus of attention during movement. This phenomenon, referred to as attentional focus, comprises two types of attention: internal focus (IF) and external focus (EF) (Wulf et al., 2010; Sawai et al., 2022a). IF refers to attention focused on a body part, such as the hand or foot, whereas EF refers to attention directed toward the external environment, such as a pointing cursor or an item. Many previous studies have reported that EF enhances performance compared with IF when the same postural control task is performed under both IF and EF conditions (Park et al., 2015). The effectiveness of the EF in healthy younger adults has been confirmed in a postural holding task on an unstable board (Chiviacowsky et al., 2010) and using a dynamic postural control task (Wulf et al., 2004). The effectiveness of EF in postural control in older and younger adults has also been reported (Chen et al., 2023). Although several studies have demonstrated the effectiveness of EF in postural control, we found individual dominance of performance-enhancing attentional focus in standing postural control in healthy younger adults (Sawai et al., 2022b, 2023). This suggests that there is an IF-dominant group with high IF performance and an EF-dominant group with high EF performance. Nevertheless, the dominance of attentional focus in standing postural control in older adults, whose cognitive and attentional functions are reduced compared to younger adults (Lacour et al., 2008), has not been clarified. Elucidating this, standing postural control training that takes into account the dominance of attentional focus could be devised to prevent falls in older adults.

Therefore, this study had the following objectives: To assess (i) the dominance of attentional focus in standing postural control in healthy younger and older adults and (ii) the relationship between attentional function and standing postural control performance under IF and EF conditions. In this study, we hypothesized that the dominance of attentional focus in standing postural control would be confirmed in older adults as well as in younger adults. In addition, since attentional function declines with age (Lacour et al., 2008), we expected that standing postural control performance under attentional focus conditions, particularly in older individuals, would be affected.

## 2 Materials and methods

### 2.1 Participants

Thirty-one healthy younger adults under 26 years of age (age: 21.71 ± 0.46 years, height: 164.48 ± 8.42 cm, body weight: 56.44 ± 12.56 kg) and 31 healthy older adults over 65 years of age (age: 73.52 ± 4.41 years, height: 156.87 ± 7.21 cm, body weight: 48.99 ± 15.32 kg) were recruited in this study. The healthy younger participants group included 11 men and 20 women, while the healthy older participants group comprised five men and 26 women. The inclusion criteria were: Healthy participants (i) with normal or corrected-to-normal vision; (ii) without fractures, injuries, lacerations, or motor paralysis limiting limb mobility; and (iii) with the ability to stand and walk without assistance. The Japanese version of the Rapid Dementia Screening Test (Adachi et al., 2021) was administered to older adults; participants with a score of < 8 points were excluded on suspicion of cognitive impairment (Kalbe et al., 2003). The sample size was determined using WebPower and R (Zhang and Yuan, 2018). With an effect size of 0.30, Numerator degree of freedom = 1.00, α = 0.05, and power (1 – β) = 0.80 at a confidence level of 95%, the determined sample size for both the two-factor analysis of variance (ANOVA) and the three-factor ANOVA was 45. All participants provided informed consent, and the study was conducted in accordance with the Declaration of Helsinki. This study was approved by the Institutional Ethics Committee of Kyoto Tachibana University (approval no. 23-59).

### 2.2 Study protocol

**Figure 1:**
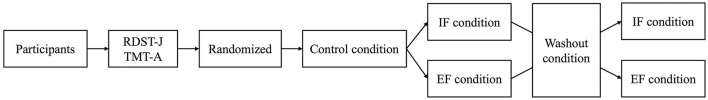
Study protocol. This study employed a randomized crossover design. The participants were asked to perform the TMT-A, followed by a standing postural control task. The standing postural control task was performed in a randomized sequence under the IF and EF conditions after the control condition. A washout condition was set between the IF and EF conditions. TMT-A, trail-making test part A; IF, internal focus; EF, external focus.

This study employed a randomized crossover design (Figure 1). Initially, the participants were evaluated using the Trail Making Test Part A (TMT-A). Next, the participants were instructed to perform a standing postural control task under the control condition, followed by tasks under the IF and EF conditions, in a randomized sequence. To ensure that the preceding condition did not influence the subsequent one, a washout condition similar to the control condition was established between the tasks under the IF and EF conditions.

### 2.3 Measures

The attentional function was evaluated using the TMT-A (Spreen and Strauss, 1998; Tombaugh, 2004). Using the TMT-A, the participants were required to connect numbers 1–25 that were randomly placed on paper in an ascending order as quickly as possible. The time taken to complete the task was measured in seconds, with shorter times indicating superior attentional function.

**Figure 2:**
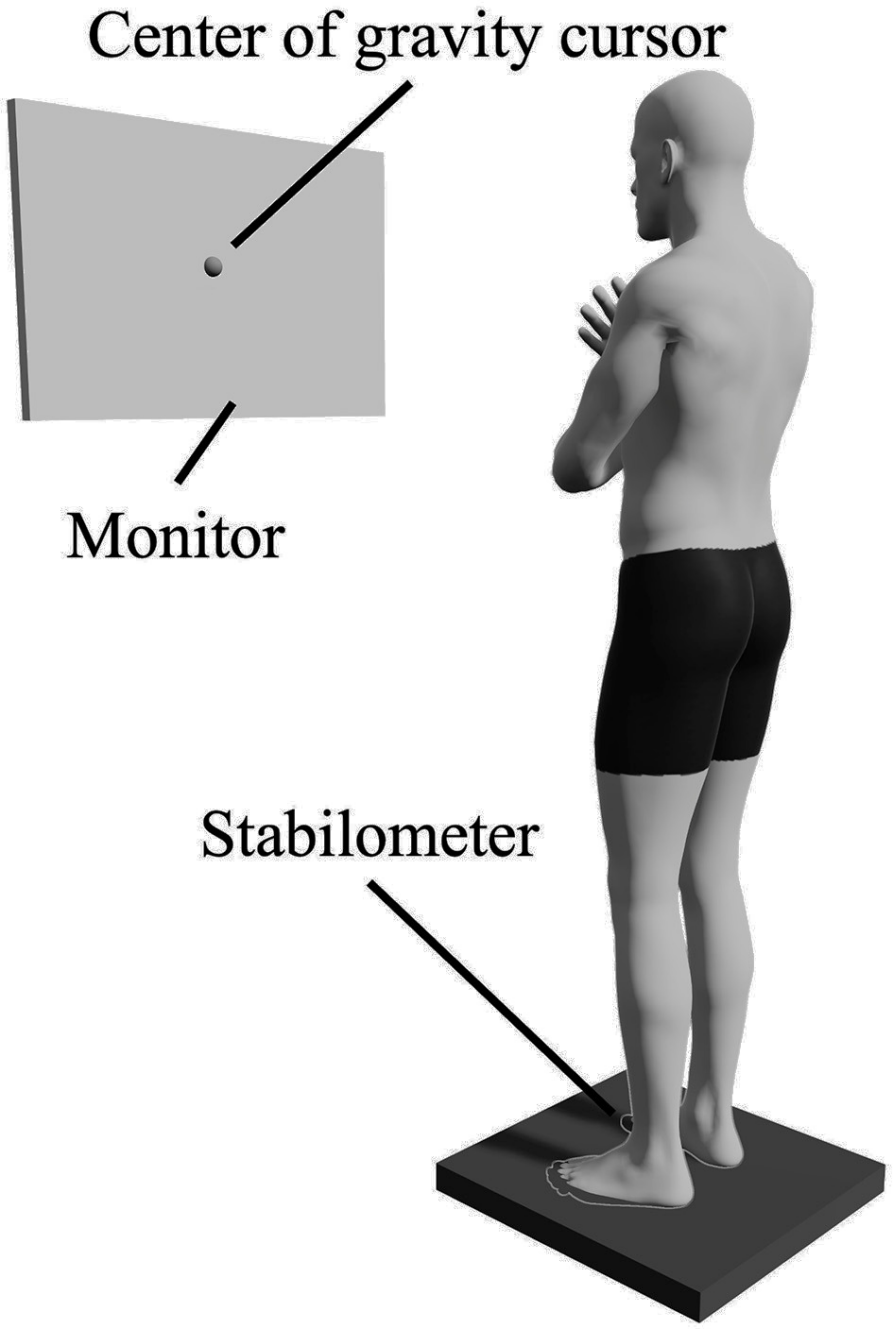
Experimental setup. The participants were instructed to stand barefoot with their arms crossed over their chests on the stabilometer. A monitor was placed in front of the participant at eye level, and it displayed the center of gravity cursor in real time.

**Figure 3:**
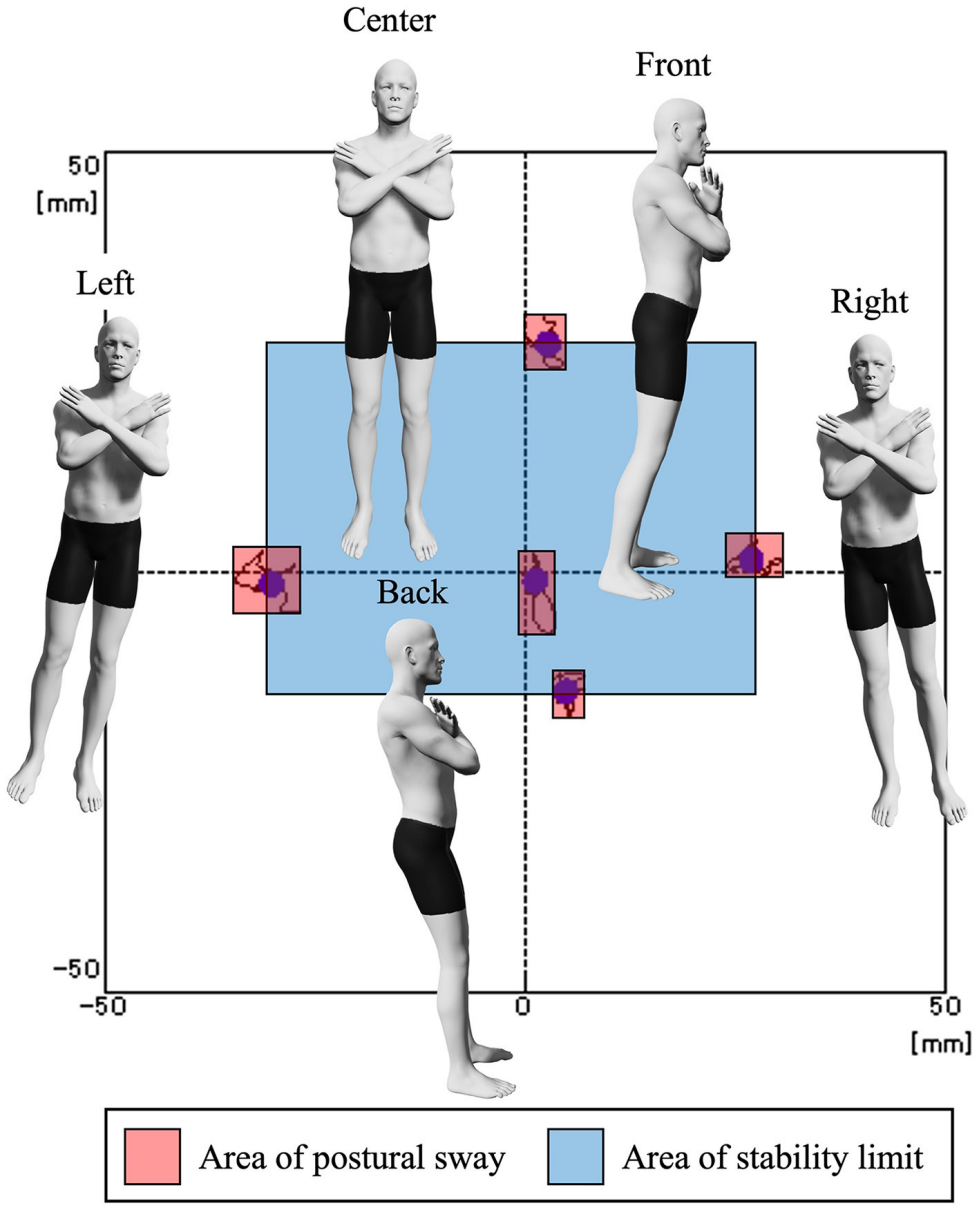
Index of postural stability. The participants were first examined for the sway of the center of gravity at the center position. Next, the participants had to hold a posture in which the center of gravity was shifted maximally to the front, back, right, and left. The area of postural sway and the area of stability limit were calculated from the center of gravity sway data in five directions, and the IPS was calculated.

The index of postural stability (IPS) (Suzuki et al., 2018; Sawai et al., 2022b, 2023) was used to assess standing postural control ability. For this purpose, the participants were asked to stand barefoot on a stabilometer (T.K.K. 5810; Takei Kiki Kogyo Co., Ltd., Niigata, Japan) with their arms crossed in front of their chests. The stabilometer measured the sway of the center of gravity within an area of 360 mm × 360 mm. The sampling rate was set to 100 Hz. Monitors were placed at 1.5 m in front of the participant, such that their centers were at the eye level of the participants. The center-of-gravity cursor, as measured by the stabilometer, was projected in real-time (Figure 2). Regarding the IPS measurements, the sway of the center of gravity was first measured for 10 s in the center position. Then, the center of gravity was held in a posture with maximum movement to the front, back, left, and right, and the sway of the center of gravity was measured for 10 s in each direction. The areas of postural sway and stability limit were calculated from the measured center-of-gravity sway data in five directions (Figure 3). IPS was calculated using the following equation:


$$\begin{array}{l} IPS=\log\frac{Area\;of\;stability\;limit+Area\;of\;postural\;sway}{Area\;of\;postural\;sway} \end{array}$$

Here, the “area of postural sway” was defined as the average of the rectangular area of the center-of-gravity sway in each direction, serving as an indicator of the ability to hold the center-of-gravity in a fixed position. The “area of stability limit” was calculated using the formula “distance between front and rear center-of-gravity movement of anterior and posterior positions × distance between right and left center-of-gravity movement of right and left positions.” The area of the stability limit reflected the ability to move the center of gravity within the base of the support. A high IPS value also implied a high-standing postural control performance.

In this study, the target of attention was manipulated by the verbal instructions for each condition. Under the control and washout conditions, the verbal instruction was “lean front (back, right, left) and try not to move as much as possible,” without reference to the object of attention. Under the IF condition, attention was focused on the foot with the instruction, “Pay attention to the weight on the foot; put your weight on the front (back, right, left) of the foot and try not to move it as much as possible.” In contrast, under the EF condition, the participant's attention was focused on the center-of-gravity cursor on the monitor with the instruction, “Pay attention to the center-of-gravity cursor projected on the monitor, move the point up (down, right, left) and try not to move it as much as possible” (Sawai et al., 2022b, 2023). Immediately after the IF and EF conditions, the participants self-evaluated their ability to focus their attention as per the verbal instruction on a numerical rating scale (0–100). The participants who scored < 60 were considered not to have focused their attention as per the verbal instruction and were consequently excluded from the analysis (Richer et al., 2017; Sawai et al., 2022b, 2023).

Based on the IPS results, the participants who achieved a higher IPS under the IF condition than under the EF condition were defined as the IF-dominant group. Conversely, the participants who achieved a higher IPS under the EF condition than under the IF condition were defined as the EF-dominant group (Sakurada et al., 2019b; Sawai et al., 2022b, 2023).

### 2.4 Statistical analyses

First, a chi-square test was used to compare the sex ratio between younger and older adults. After that, the normality of all data was confirmed using the Shapiro–Wilk test. The IPS was then compared using a mixed-design 2-way analysis of variance (ANOVA) using two factors: age (younger adults, older adults) and condition (IF condition, EF condition). In addition to this, the IPS for each condition was compared using a mixed-design 3-way ANOVA using three factors: attentional focus dominance (IF-dominant group, EF-dominant group), age (younger adults, older adults), and condition (IF condition, EF condition). We conducted a chi-square test to compare the distribution of participants based on the order of conditions performed (IF condition first, EF condition first) and the dominance of attentional focus (IF-dominant group, EF-dominant group). Furthermore, the TMT-A times of participants were compared using a 2-way ANOVA with two factors: dominance of attentional focus (IF-dominant group, EF-dominant group) and age (younger adults, older adults). Bonferroni *post-hoc* tests were used for multiple comparisons of all ANOVAs. The relationship between the IPS and TMT-A times for each condition was examined using Pearson's correlation analysis separately for the younger IF-dominant group, the younger EF-dominant group, the older IF-dominant group, and the older EF-dominant group. SPSS version 29.0 was used for statistical analysis. The statistical significance level was set at 5%.

## 3 Results

The chi-square test found no significant differences in the number of male and female participants between younger and older adults (χ^2^ = 3.03, *p* = 0.08).

The Shapiro–Wilk test showed that all data were normally distributed (*p* > 0.05). Comparing the IPS under the IF and EF conditions between younger and older adults, there was no significant interaction between the two factors of age and condition (F = 1.52, partial η^2^ = 0.03, *p* = 0.22). There was a significant main effect of age (F = 42.64, partial η^2^ = 0.42, *p* < 0.01). The *post-hoc* test results showed that the IPS under both the IF and EF conditions was significantly higher among younger adults than among older adults (*p* < 0.01). However, there was no significant main effect of the condition (F = 2.45, partial η^2^ = 0.04, *p* = 0.12) (Table 1).

**Table 1:** Comparison of IPS under the IF and EF conditions between younger and older adults.

	**IF condition**	**EF condition**	**Age** × **condition**		**Age**		**Condition**	
			**F-value**	***P*-value**	**F-value**	***P*-value**	**F-value**	***P*-value**
Younger adults	2.18 ± 0.22	2.17 ± 0.21	1.52	0.22	42.64	< 0.01	2.45	0.12
Older adults	1.81 ± 0.29	1.74 ± 0.31						

IPS was significantly higher in younger adults compared with older adults under both IF and EF conditions (p < 0.05). IF, internal focus; EF, external focus; IPS, index of postural stability.

**Figure 4:**
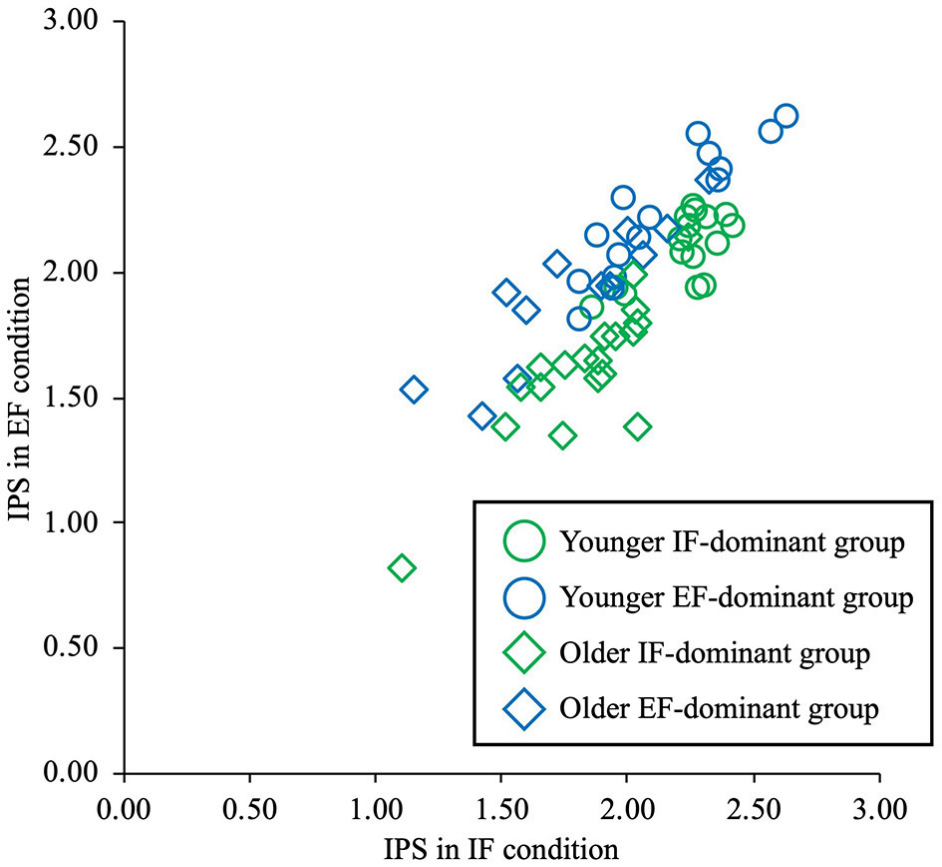
The dominance of attentional focus in younger and older adults. The vertical axis shows the IPS values under the EF condition, and the horizontal axis shows the IPS values under the IF condition. The round plots represent data for younger people, and the diamond plots for older adults. The green plot shows the IF-dominant group and the blue plot shows the EF-dominant group. The participants were divided into IF-dominant groups with high IPS under the IF condition and EF-dominant groups with high IPS under the EF condition for both younger and older adults. IPS, index of postural stability; IF, internal focus; EF, external focus.

**Figure 5:**
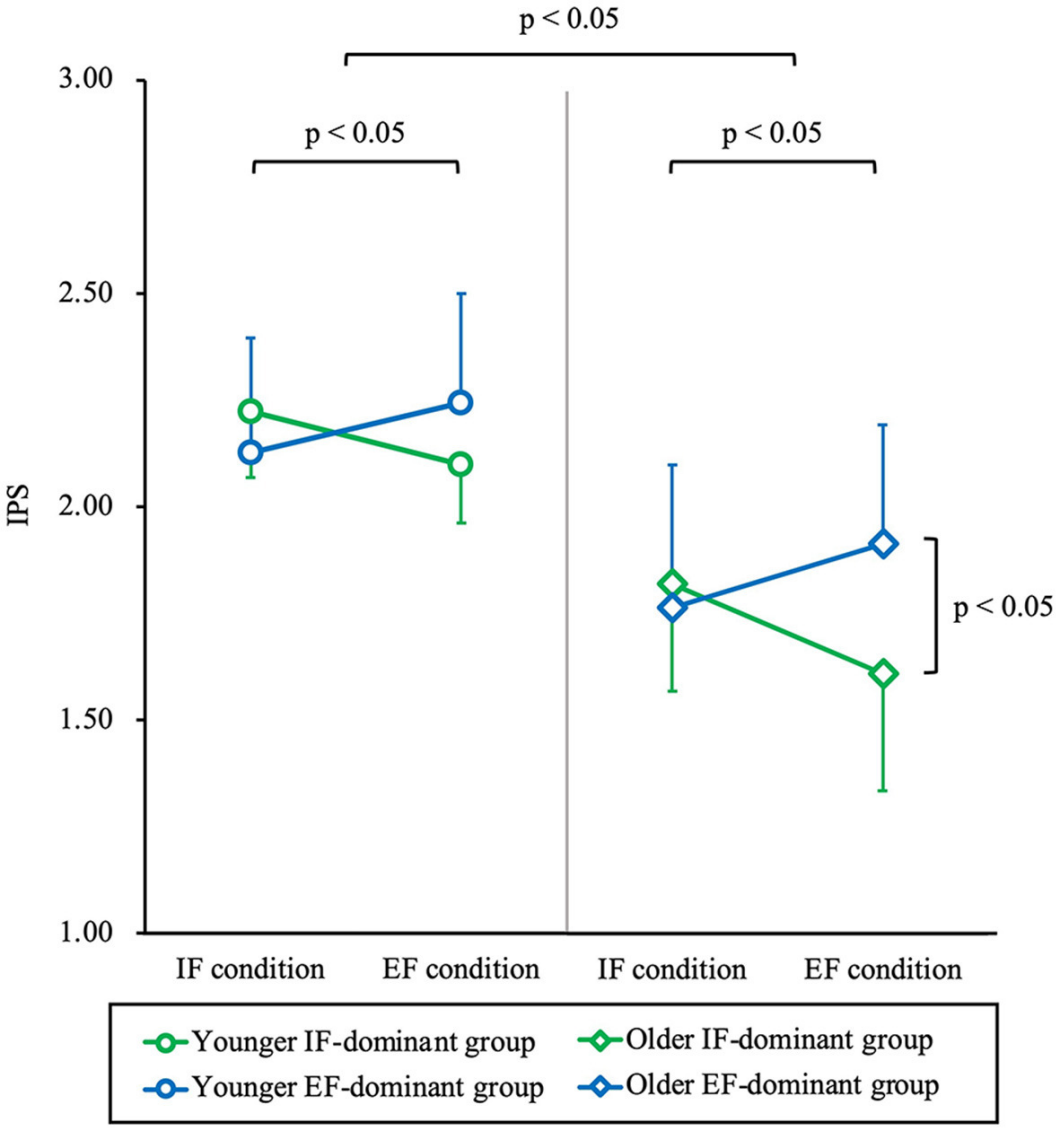
Comparison of IPS between younger and older adults, IF-dominant and EF-dominant group, and IF and EF condition. The vertical axis shows IPS. The round plots represent data for younger people, and the diamond plots for older adults. The green plot shows the IF-dominant group and the blue plot shows the EF-dominant group. IPS was significantly lower among the older adults compared to the younger adults (*p* < 0.05). In both younger and older adults, the IF-dominant group had a significantly higher IPS under the IF condition than under the EF condition, and the EF-dominant group had a significantly higher IPS under the EF condition than under the IF condition (*p* < 0.05). Furthermore, IPS under the EF condition was significantly higher in the EF-dominant group than in the IF-dominant group among older adults (*p* < 0.05). IPS, index of postural stability; IF, internal focus; EF, external focus.

The IPS under the IF and EF conditions was compared and grouped into IF-dominant and EF-dominant groups. Among the younger adults, 16 belonged to the IF-dominant group and 15 to the EF-dominant group. In comparison, among the older adults, 19 belonged to the IF-dominant group and 12 to the EF-dominant group (Figure 4). A comparison of the IPS with a 3-way ANOVA revealed no significant interaction between the three factors of age, dominance of attentional focus, and condition (F = 3.07, partial η^2^ = 0.05, *p* = 0.09). Similarly, there were no significant interactions between age and dominance of attentional focus (F = 0.65, partial η^2^ = 0.01, *p* = 0.42) or between age and condition (F = 0.59, partial η^2^ = 0.01, *p* = 0.45). However, a significant interaction was observed between the dominance of attentional focus and condition (F = 77.58, partial η^2^ = 0.57, *p* < 0.01). *Post hoc* tests showed that the IF-dominant group had a significantly higher IPS under the IF condition than that under the EF condition; the EF-dominant group had a significantly higher IPS under the EF condition than that under the IF condition for both younger and older adults (*p* < 0.01). In addition, the IPS under the EF condition was significantly higher in the EF-dominant group than in the IF-dominant group (*p* < 0.01). Age had a significant main effect (F = 38.75, partial η^2^ = 0.40, *p* < 0.01). *Post-hoc* tests showed that the IPS of both the IF-dominant and EF-dominant groups was significantly higher among younger individuals than among older individuals under both the IF and EF conditions (*p* < 0.01) (Figure 5). No significant differences in numbers were detected between the IF-dominant and EF-dominant groups when comparing the numbers of participants who performed the IF condition first and those who performed the EF condition first, both in younger (χ^2^ = 0.78, *p* = 0.38) and older adults (χ^2^ = 2.62, *p* = 0.11).

**Figure 6:**
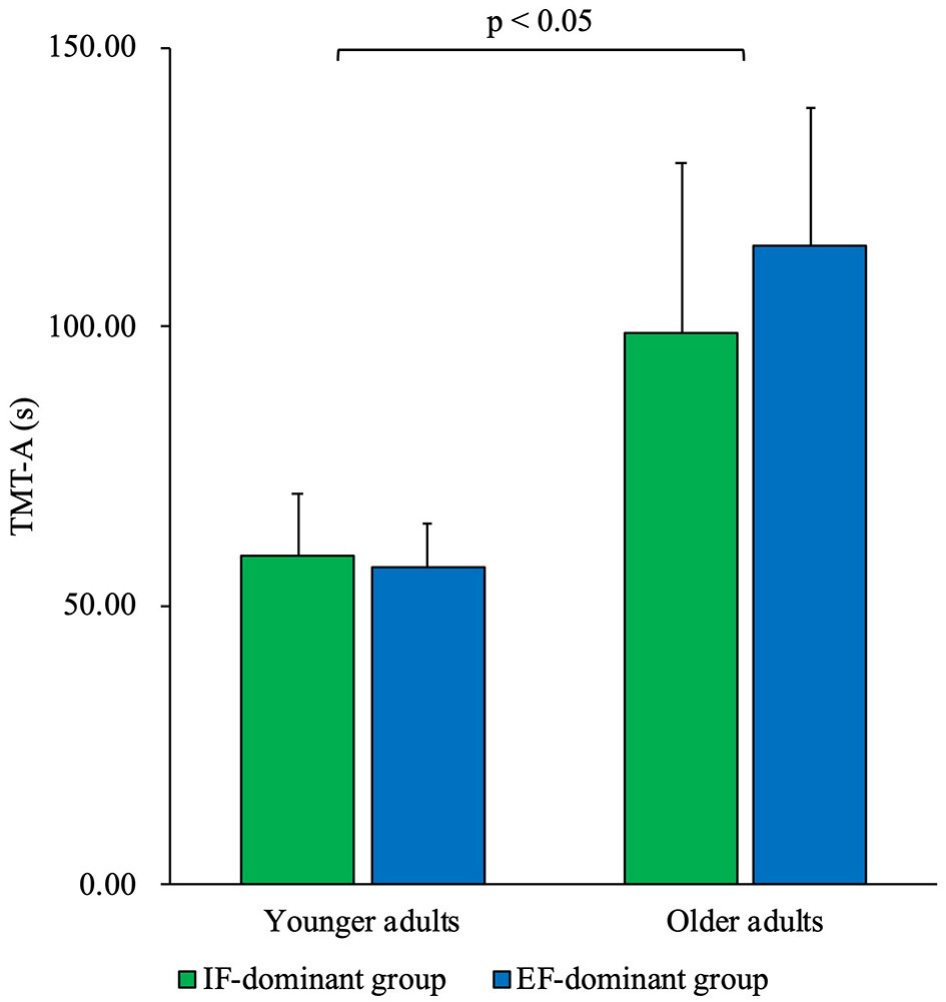
The comparison of time required to complete TMT-A between younger and older adults and between IF-dominant and EF-dominant groups. The vertical axis shows the time required for TMT-A. The green bar represents the IF-dominant group, and the blue bar the EF-dominant group. The older adults took significantly longer to complete the TMT-A compared to the younger adults (*p* < 0.05). However, there were no significant differences in the time required to complete the TMT-A between the IF-dominant and EF-dominant groups for both younger and older adults (*p* > 0.05). TMT-A, trail-making test part A; IF, internal focus; EF, external focus.

Comparison of TMT-A times between the groups using 2-way ANOVA showed no significant interaction between the two factors of age and dominance of attentional focus (F = 2.65, partial η^2^ = 0.04, *p* = 0.11). Additionally, the age factor had a significant main effect on TMT-A time (F = 80.30, partial η^2^ = 0.58, *p* < 0.01). The *post-hoc* test showed that the required TMT-A time was longer in older adults than in younger adults (*p* < 0.01). However, there was no significant main effect of the dominance factor on attentional focus (F = 1.47, partial η^2^ = 0.03, *p* = 0.23) (Figure 6).

**Figure 7:**
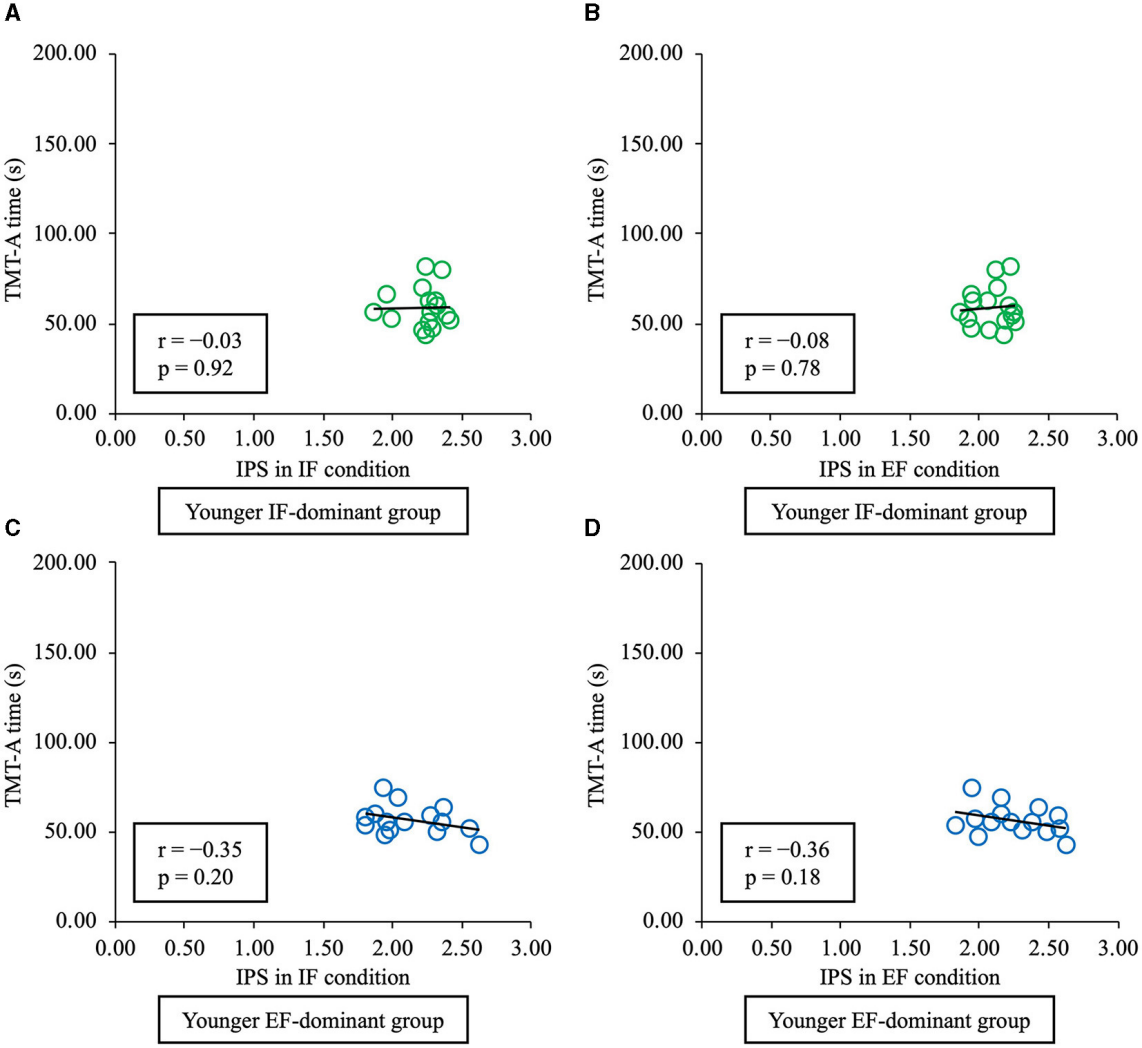
The relationship between time required to complete TMT-A and IPS in healthy younger adults. The vertical axis shows the time required for TMT-A, and the horizontal axis shows the IPS for each condition. The green plot represents the IF-dominant group, and the blue plot the EF-dominant group. **(A)** In the IF-dominant group, IPS under the IF condition did not show a significant correlation with TMT-A time (*p* > 0.05). **(B)** In the IF-dominant group, IPS under the EF condition did not show a significant correlation with TMT-A time (*p* > 0.05). **(C)** In the EF-dominant group, IPS under the IF condition did not show a significant correlation with TMT-A time (*p* > 0.05). **(D)** In the EF-dominant group, IPS under the EF condition did not show a significant correlation with TMT-A time (*p* > 0.05). TMT-A, trail-making test part A; IPS, index of postural stability; IF, internal focus; EF, external focus.

**Figure 8:**
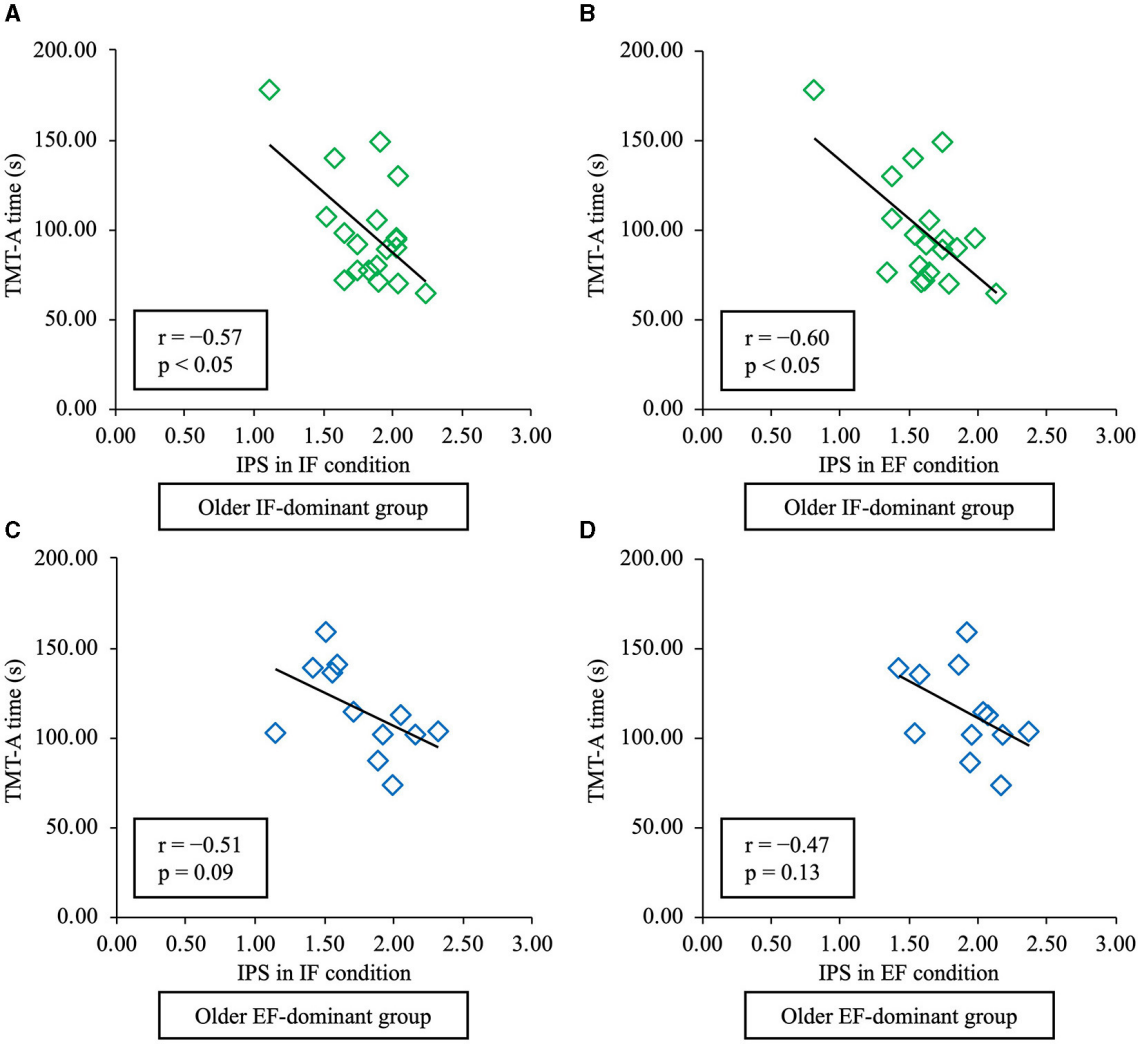
Relationship between time required to complete TMT-A and IPS in healthy older adults. The vertical axis shows the time required for TMT-A, and the horizontal axis shows the IPS for each condition. The green plot represents the IF-dominant group, and the blue plot the EF-dominant group. **(A)** In the IF-dominant group, IPS under the IF condition showed a significant negative correlation with TMT-A time (*p* < 0.05). **(B)** In the IF-dominant group, IPS under the EF condition showed a significant negative correlation with TMT-A time (*p* < 0.05). **(C)** In the EF-dominant group, IPS under the IF condition did not show a significant correlation with TMT-A time (*p* > 0.05). **(D)** In the EF-dominant group, IPS under the EF condition did not show a significant correlation with TMT-A time (*p* > 0.05). TMT-A, trail-making test part A; IPS, index of postural stability; IF, internal focus; EF, external focus.

The relationship between the IPS and TMT-A time in each condition was assessed using Pearson's correlation analysis, which showed that the TMT-A time in younger people was not significantly correlated with IPS under the IF (r = −0.03, *p* = 0.92) or EF conditions (r = −0.08, *p* = 0.78) in the IF-dominant group and under the IF (r = −0.35, *p* = 0.20) or EF (r = −0.36, *p* = 0.18) condition in the EF-dominant group (Figure 7). In contrast, in the older IF-dominant group, there was a significant negative correlation between the TMT-A time and IPS under the IF condition (r = −0.57, *p* = 0.01) and between the TMT-A time and IPS under the EF condition (r = −0.60, *p* < 0.01). However, there was no significant correlation between the IPS and TMT-A time under IF (r = −0.51, *p* = 0.09) and EF condition (r = −0.47, *p* = 0.13) in the EF-dominant group (Figure 8). Correlation analysis in older adults showed a medium correlation between IPS under the IF and EF conditions and TMT-A time taken in both IF-dominant and EF-dominant groups.

## 4 Discussion

This study examined the dominance of attentional focus in standing postural control in healthy younger and older adults and the relationship between the dominance of attentional focus and TMT-A time. The results showed that the performance of standing postural control was lower in older adults than in younger adults. However, older adults showed the same attentional focus dominance as did younger adults. Additionally, among older adults, IPS under the IF condition remained stable, regardless of the dominance of attentional focus. However, IPS under the EF condition was notably lower in the IF-dominant group compared with the EF-dominant group. Therefore, it was suggested that performance under the EF condition, which exhibited more variability among individuals than under the IF condition, might have influenced the dominance of attentional focus in older adults. These results suggest that interventions based on attentional focus dominance may enhance standing postural control in older adults. Particularly, in the older IF-dominant group, EF condition interventions decreased standing postural control performance, implying that intervention under the IF condition according to attentional focus dominance may be preferable.

### 4.1 Effects of attentional focus on standing postural control in younger and older adults

We found that IPS was significantly lower in healthy older adults compared with healthy younger adults. IPS is a standing postural control assessment index that is unlikely to cause a ceiling effect. It has been reported to decline rapidly, particularly after the age of 60 years (Suzuki et al., 2018). In addition, the IPS for 19–25-year-olds and 66–75-year-olds were reported to be 2.08 ± 0.19 and 1.63 ± 0.36, respectively (Suzuki et al., 2018); the results of the present study are similar to those of the aforementioned study. Our finding of a lower IPS among older adults than in younger adults may be attributed to the fact that the ability to control standing posture declines with age (Stoffregen, 2016). Furthermore, IPS has been reported to be heavily influenced by vision and plantar superficial sensation (Suzuki et al., 2018). Visual information processing (Zhang et al., 2008) and plantar sensory processing function (Peters et al., 2016) decline with age. Therefore, it is possible that the IPS in older adults in this study was lower than that in younger adults.

There were no significant differences between the IPS in the younger and older adults under the IF and EF conditions. This result indicates that performance is not necessarily higher under the EF condition than that under the IF condition. Many previous studies have shown that performance is better under the EF condition than under the IF condition (Wulf et al., 2010; Sawai et al., 2022a), and similar results have been reported for postural control (Chiviacowsky et al., 2010). However, we found that there was an individual dominance in the optimal attentional focus condition for standing postural control in healthy younger adults (Sawai et al., 2022b, 2023). This implies that there is an IF-dominant group that performs better under the IF condition and, similarly, an EF-dominant group that performs better under the EF condition. As the optimal attentional focus condition differs between individuals, it is possible that high performance under the EF condition was not observed in this study, as has been reported in many previous studies.

### 4.2 The dominance of attentional focus in standing postural control in younger and older adults

The participants in this study were divided into IF-dominant and EF-dominant groups for both healthy younger and older adults. In previous studies, we reported the dominance of attentional focus in standing postural control in healthy younger adults (Sawai et al., 2022b, 2023). The dominance of attentional focus has also been confirmed in an upper limb tracking task in healthy younger adults (Sakurada et al., 2016, 2019a,b, 2022). Therefore, the results of this study indicated that the dominance of attentional focus in standing postural control existed not only in younger adults but also in older adults.

In both the IF-dominant- and EF-dominant groups, the IPS under the IF and EF conditions was lower in older adults than in younger adults. These results indicate that the IPS is affected by age-related decline in the ability to control standing posture, irrespective of the dominance of attentional focus. The ability to control standing posture is reduced in older adults (Stoffregen, 2016), and this is not only due to muscle weakness (Gouveia et al., 2020) but also to a decrease in visual information processing (Zhang et al., 2008) and plantar sensory processing (Peters et al., 2016). Differences in sensory processing characteristics have been reported to exist between the IF-dominant and EF-dominant groups, with superficial sensory processing being prioritized in the IF-dominant group and visual information processing in the EF-dominant group (Sakurada et al., 2022). Both visual information and plantar sensory processing abilities decline with age, which may have led to a lower IPS in older adults than in younger adults in both the IF-dominant and EF-dominant groups. Furthermore, electroencephalography activity in the frontal and parietal lobes is involved in the dominance of attentional focus in standing postural control in young adults (Sawai et al., 2022b). On the other hand, it has been pointed out that older adults had higher electroencephalography activity and mobilize more cortex during postural control than did younger adults (Rubega et al., 2021). This implies that older adults perform effortful postural control by excessive neuronal mobilization. Such changes in neurological strategies for postural control may influence the decreased ability to control standing posture in older adults. Therefore, it is possible that the IPS of the older adults in both the IF-dominant and EF-dominant groups was lower than that of the younger adults in this study as well.

#### 4.2.1 The dominance of attentional focus in standing postural control in younger adults

In this study, there was a significant interaction between the factors of condition and dominance of attentional focus. However, there were no significant group differences between the IF-dominant and EF-dominant groups in the IPS under the IF and EF conditions among younger adults. This result indicated that the effect of attentional focus on the IPS might be smaller in the participants with high-standing postural control ability, such as younger adults. In a previous study, it was reported that there was no difference in performance between the IF and EF conditions on easy tasks but that the difference in performance between the IF and EF conditions was more apparent on difficult tasks (Wulf et al., 2007). Thus, attentional focus was found to be more effective in more difficult tasks. It has also been reported that postural control in young adults is carried out by subcortical automatic control and is not affected by the stimuli presented (Honeine et al., 2017). In this study, younger adults had higher standing postural control ability compared with older adults, reducing the difficulty level in the IPS. Therefore, it is possible that the impact of attentional focus on the IPS was small, leading to no significant difference in IPS between the IF-dominant and EF-dominant groups under the IF and EF conditions among younger adults.

#### 4.2.2 The dominance of attentional focus in standing postural control in older adults

Under the IF condition, there were no significant differences in IPS between the IF-dominant and EF-dominant groups in older adults. However, under the EF condition, the IPS was significantly higher in the EF-dominant group than that in the IF-dominant group. This result suggests that performance under the IF condition is independent of the dominance of attentional focus in older adults and that the dominance of attentional focus may influence performance under the EF condition. This means that older adults with a low IPS under the EF condition were in the IF-dominant group, whereas those with a high IPS under the EF condition were in the EF-dominant group.

Compared with younger adults, older adults tend to perform postural control with proprioceptive information that is superior to visual and vestibular sensory information (Wiesmeier et al., 2015). In this study, the participants were asked to focus their attention on their feet under the IF condition, which promotes standing postural control with superficial sensory and proprioceptive superiority (Gottwald et al., 2020). Due to the attention to the proprioceptive senses that older adults tended to use under the IF condition, the IPS under the IF condition may have remained constant, independent of the dominance of attentional focus. Therefore, it is possible that there was no significant difference in IPS under the IF condition between the IF-dominant- and EF-dominant groups among older adults.

Visual information processing has been shown to decline with age (Ebaid and Crewther, 2019). Furthermore, there are individual differences in visual information processing in older adults (Owsley, 2012). A correlation between motor perception and postural control in older adults with respect to visual information processing has been reported (Wood et al., 2022). Thus, it is clear that visual information processing, which declines with age, influences postural control. Under the EF condition in this study, attention was focused on the center of gravity on the monitor, which promoted visual information-dominant postural control. Therefore, the finding that the IPS in the IF-dominant group was significantly lower than that in the EF-dominant group under the EF condition among older adults may have been attributed to the individual differences in visual information processing. In conclusion, our results suggested that performance under the EF condition but not under the IF condition could influence the dominance of attentional focus in standing postural control among older adults. On the other hand, it has been reported that in healthy older adults, postural control was not impaired by the presentation of confusing visual information during standing postural control (Pelosin et al., 2018). Thus, the effects of visual information processing on standing postural control in older adults need to be more consistent. Further research is needed to clarify the factors that influence the performance of standing postural control under the EF condition in older adults.

### 4.3 The relationship between the dominance of attentional focus in standing postural control and attentional function in younger and older adults

The time required for the TMT-A was longer in older adults than in younger adults. However, there was no significant difference between the IF-dominant- and EF-dominant groups. The time required for TMT-A has been reported to increase with age (Hashimoto et al., 2006; Periáñez et al., 2007), and it is possible that the time required for TMT-A was similarly affected by aging in this study, with older adults having a longer TMT-A time compared with younger adults. The TMT-A time may not be a major influencing factor for the dominance of attentional focus, as there were no significant differences between the IF-dominant and EF-dominant groups among either younger or older adults. Previous studies examining the factors associated with the dominance of attentional focus have found that motor imagery characteristics (Sakurada et al., 2019b) and primary somatosensory cortex responses to visual and superficial sensations (Sakurada et al., 2022) are relevant. This suggests that individual characteristics in the processing of sensory-motor information are primarily related to the dominance of attentional focus and that TMT-A time may not be a major associated factor.

The association between IPS under each condition and the time required for TMT-A was examined in each group; no significant correlation was found between IPS and TMT-A time in younger adults. TMT-A is a test of general cognitive and attentional functions, reflecting visual search and scanning abilities and complex attentional functions (Robins Wahlin et al., 1996; Allen and Haderlie, 2010). Cognitive and attentional functions bear no influence on standing postural control in younger adults with high performance compared with older adults (Bernard-Demanze et al., 2009). Therefore, there may have been no association between the TMT-A time and IPS under the IF and EF conditions among younger adults with higher standing postural control performance.

In contrast, among older adults, the IPS in the IF-dominant group under IF and EF conditions showed a significant correlation with TMT-A time. In contrast, the IPS in the EF-dominant group showed no significant correlation with TMT-A time. Considering that the correlation analysis in this study was a subgroup analysis and the number of evaluated participants was small, it can be inferred that the results could be exploratory. Therefore, interpreting the results by focusing on the correlation coefficient rather than on whether a significant correlation was detected is necessary. Based on the above, a medium correlation was found between IPS under the IF and EF conditions and TMT-A in both the older IF-dominant and the older EF-dominant groups. The results indicated that there was an association between standing postural control performance and attentional function in older adults, regardless of attentional focus and the dominance of attentional focus. The relationship between postural control and attentional function has been verified in many studies. Moreover, it has been reported that older adults could have poorer performance in postural control during dual tasks that required attentional demands (Brown et al., 1999; Woollacott and Shumway-Cook, 2002). In this study, verbal instruction to focus attention on the feet and the center of gravity point was given under each of the IF and EF conditions, and this may have demanded attention. Therefore, a medium correlation may have been found between the TMT-A, which assesses attentional function (Robins Wahlin et al., 1996), and the IPS under the IF and EF conditions.

### 4.4 Limitations

This study has some limitations. First, it assessed TMT-A time as a relevant factor for the dominance of attentional focus and failed to consider other factors. A previous study on healthy younger participants reported that differences in responses to visual and tactile information in the primary somatosensory cortex were related to the dominance of attentional focus in an upper limb tracking task (Sakurada et al., 2022). Therefore, not only did they validate TMT-A times in their study, but they also demonstrated that other indices may be related to the dominance of attentional focus in standing postural control among older adults. Future studies should examine the factors associated with the dominance of attentional focus from multiple perspectives across many outcomes. Second, although this study examined changes in performance, learning effects could not be examined. Future studies should investigate the effects of attentional focus dominance on motor learning during standing postural control to obtain more clinically useful results. Third, this study failed to take into account the participants' sporting history. A previous study reported that sports history affected the dominance of attentional focus in the upper limb tracking task (Sakurada et al., 2016). Therefore, it is possible that the participants' sporting history also influenced the results of this study. In future studies, assessing the dominance of attentional focus by asking for basic background information about the participants, such as their sporting history, may be useful. Fourth, the sex ratio of the participants in this study differed between younger and older adults. Although the comparison of the sex ratios showed no significant differences, the differences in the sex ratios may have influenced the results. Future studies could verify the results separately in male and female patients and eliminate the influence of sex to obtain more detailed results. Fifth, in this study, the dominance of attentional focus was examined by performing IPS measures under the IF and EF conditions in a crossover design. A chi-square test showed that the prior condition did not affect the dominance of attentional focus. However, the difference in IPS between the IF and EF conditions was small, and we cannot rule out the possibility that the order in which the task conditions were performed might have affected the dominance of attentional focus. Sixth, in the present study, the IPS under the IF and EF conditions were measured only once each. Therefore, it cannot be definitively determined that the results of the present study are not coincidental and reflect participant characteristics in participants with similar IPS values under the IF and EF conditions. Future research should shed light on the stationarity of the dominance of attentional focus.

### 4.5 Conclusion

Our results confirmed the dominance of attentional focus in standing postural control in healthy older adults as well as in healthy younger adults. In older adults, the IF-dominant group showed lower standing postural control performance under the EF condition than did the EF-dominant group. These results suggest that the dominance of attentional focus in standing postural control among older adults could influence their performance under the EF condition. They also indicated that standing postural control was affected by attentional function in the IF-dominant group, and this performance was impaired under the EF condition. The results of this study suggest the importance of an individually tailored intervention method based on the dominance of attentional focus for standing postural control among older adults. In particular, guiding the IF-dominant group toward the IF condition to avoid low performance in standing postural control is important, because the standing postural control of the IF-dominant group tends to diminish under the EF condition. The results of this study might be applied to prevent falls in community-dwelling older adults and to facilitate rehabilitation during hospitalization to effectively improve the standing postural control performance of older adults.

## Data availability statement

The raw data supporting the conclusions of this article will be made available by the authors, without undue reservation.

## Ethics statement

The studies involving humans were approved by Kyoto Tachibana University Ethics Screening Committee. The studies were conducted in accordance with the local legislation and institutional requirements. The participants provided their written informed consent to participate in this study.

## Author contributions

SS: Conceptualization, Data curation, Formal analysis, Funding acquisition, Investigation, Methodology, Resources, Validation, Visualization, Writing – original draft, Writing – review & editing. SM: Investigation, Resources, Writing – review & editing. YSa: Investigation, Resources, Validation, Writing – review & editing. SF: Investigation, Validation, Writing – review & editing. RY: Investigation, Validation, Writing – review & editing. YSh: Investigation, Validation, Writing – review & editing. HN: Conceptualization, Data curation, Formal analysis, Funding acquisition, Investigation, Methodology, Project administration, Resources, Supervision, Validation, Visualization, Writing – original draft, Writing – review & editing.
